# Supplementary material for: Bone Marrow Stroma-Induced Transcriptome and Regulome Signatures of Multiple Myeloma
Source: Cancers (Basel). 2022 Feb 13;14(4):927. doi: 10.3390/cancers14040927 (PMC8870223; doi:10.3390/cancers14040927)
Supplement: Supplementary file 1 [file cancers-14-00927-s001.zip › cancers-1584132-supplementary materials.pdf]

Supplementary Materials: Bone Marrow Stroma-induced Transcriptome and Regulome Signatures of Multiple Myeloma

Sebastian A. Dziadowicz, Lei Wang, Halima Akhter, Drake Aesoph, Tulika Sharma, Donald A. Adjeroh, Lori A. Hazlehurst and Gangqing Hu

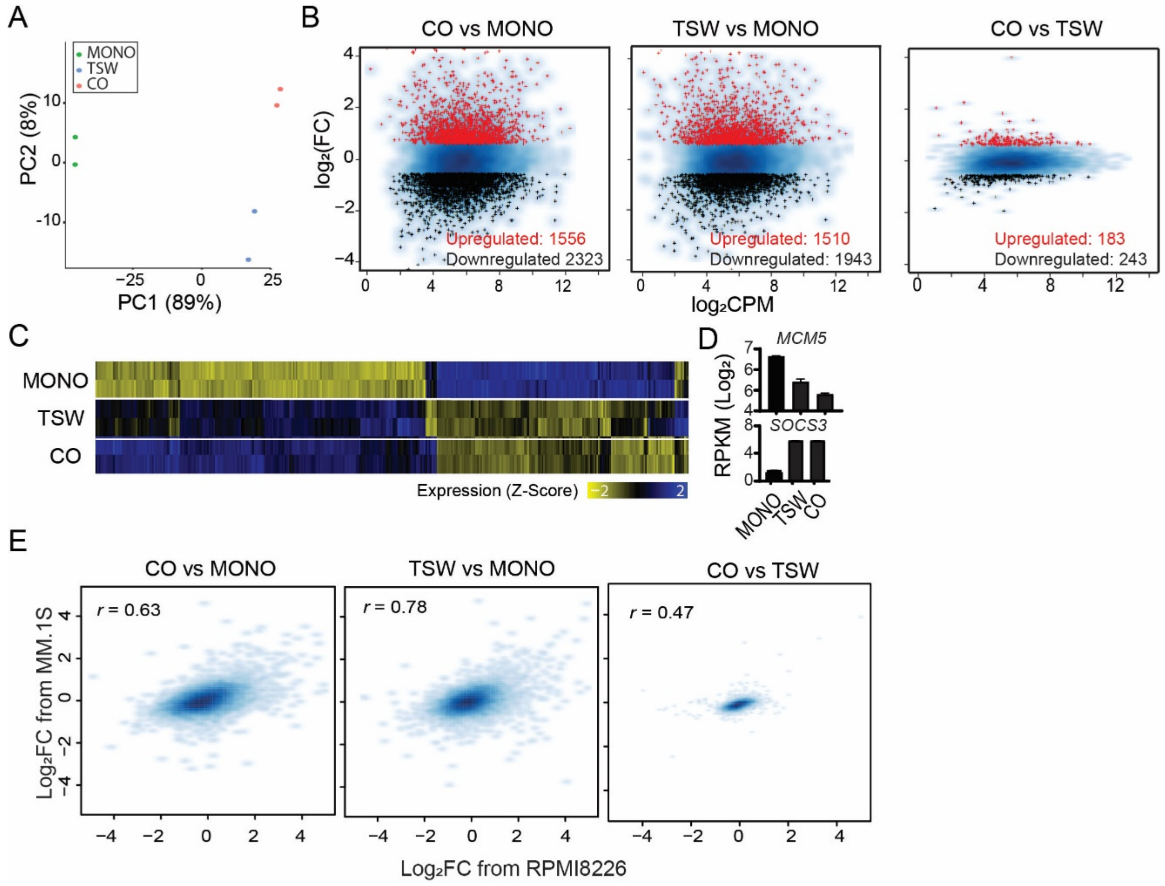

Figure S1. BMSC-induced changes in transcriptome in MM1.S are reproducible with RPMI8226. (**A**) PCA applied to RPMI8226 samples from MONO, TSW and CO based on gene expression values. TSW: MM cells in the upper transwell; CO: MM cells in the lower chamber; MONO: MM cells in monoculture. (**B**) MA-plots displaying Log_2CPM (x-axis) and fold change of expression (y-axis) for the comparisons of CO vs MONO (left), TSW vs MONO (middle), and TSW vs CO (right), all in RPMI8226. Red: genes upregulated in expression. Black: genes downregulated. Blue background: all expressed genes. (**C**) Heat map visualization of expression level for genes (columns) differentially expressed between any two from MONO, TSW, and CO from RPMI8226. Expression values were normalized into z-score for each gene across all samples. (**D**) Comparisons of gene expression level across MONO, TSW, and CO for MCM5 and SOCS3, all from RPMI8226. Error bar: standard deviation. (**E**) Smoothed scatter plot comparing FC of expression calculated from MM.1S (y-axis) and calculated from RPMI8226 (x-axis) across comparisons of CO vs MONO (left), TSW vs MONO (middle), and CO vs TSW (right). *r*: Pearson correlation coefficient.

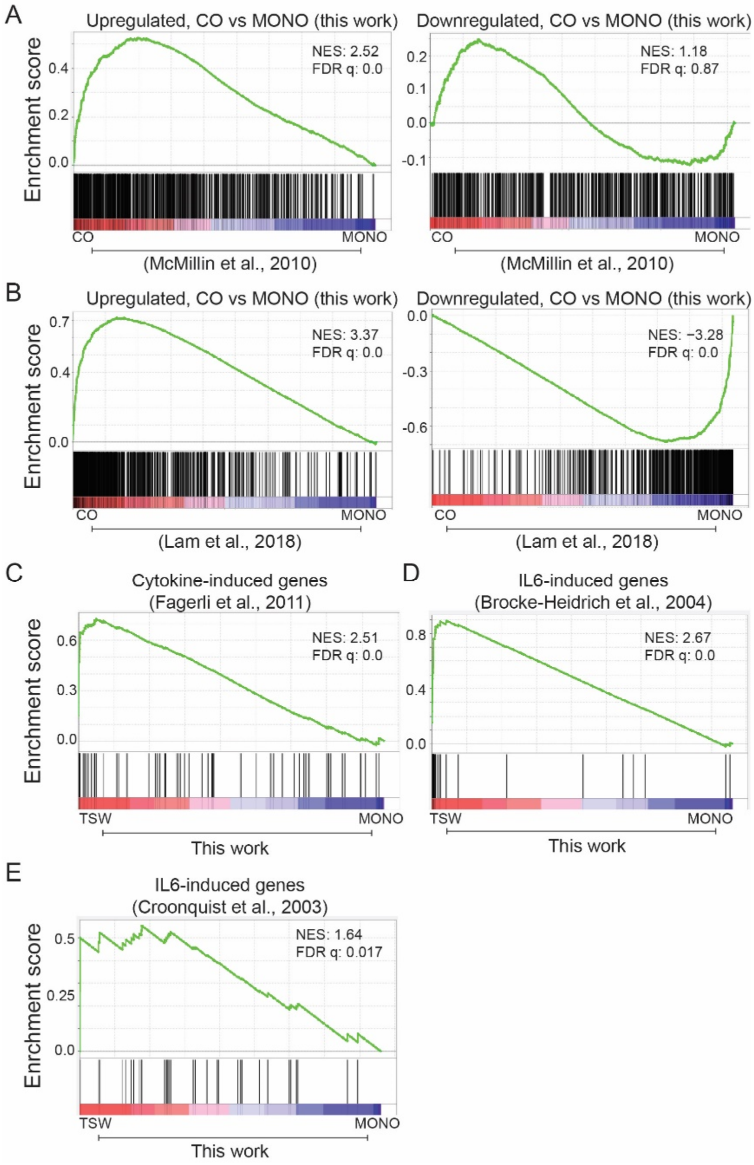

Figure S2. Validation of BMSC-induced transcription signatures with expression data sets from independent sources. (**A**) GSEA of expressed genes sorted by fold change of expression (CO/MONO; MM.1S) from high (red) to low (blue) based on expression data from McMillin et al., [1] against gene set (vertical bars) for upregulated genes (left) or downregulated genes (right) in CO vs MONO as defined in this work. CO: MM cells in the lower chamber of a coculture with BMSCs; MONO: MM.1S cells in monoculture; NES: normalization enrichment score. (**B**) GSEA of expressed genes sorted by fold change of expression (CO/MONO; MM.1S) from high (red) to low (blue) based on expression data from Lam et al., [2] against gene set (vertical bars) for upregulated genes (left) or downregulated genes (right) in CO vs MONO as defined in this work. (**C**–**E**) GSEA of expressed genes sorted by fold change of expression (TSW/MONO) from high (red) to low (blue) based on expression data from this work against gene set (vertical bars) for cytokine-induced genes by Fagerli et al. [3] (**C**), IL6-induced genes by Brocke-Heidrich et al. [4] (**D**), and IL6-induced genes by Croonquist et al. [5] (**E**). TSW: MM cells in the upper transwell of a coculture with BMSCs.

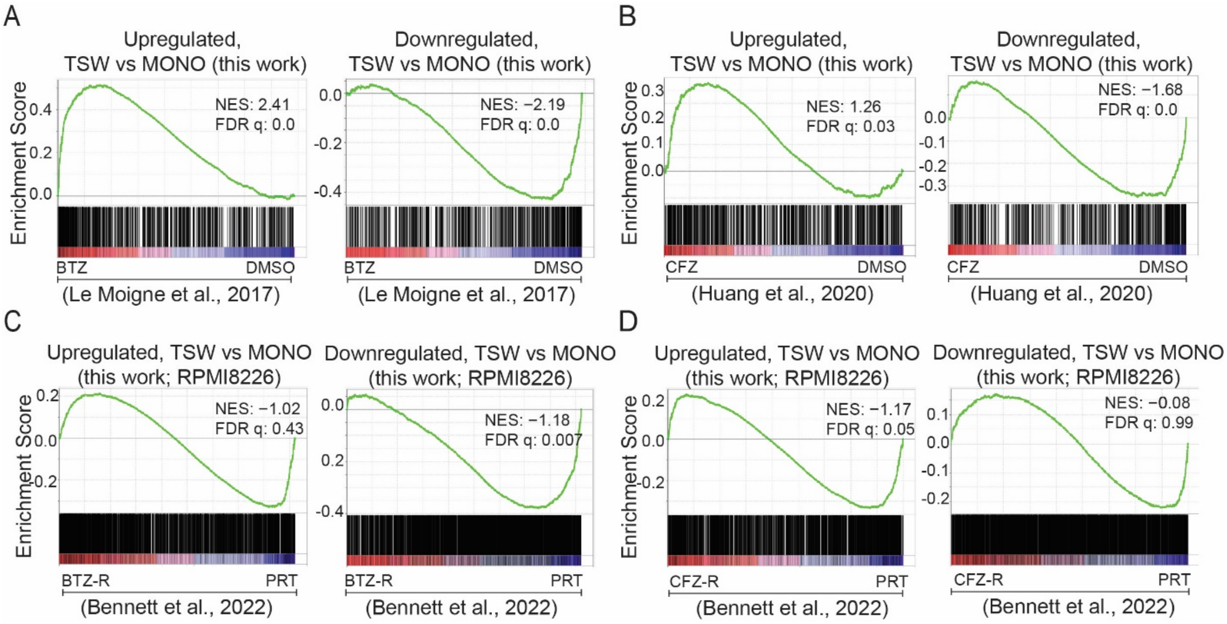

Figure S3. BMSC-induced transcription signatures recapitulated a transcription response to transient (**A&B**) but not chronic (**C**,**D**) treatment of proteasome inhibitors. (**A**) GSEA of expressed genes sorted by fold change of expression (BTZ/DMSO; MM.1S) from high (red) to low (blue) based on expression data from Le Moigne et al., [6] against gene set (vertical bars) for upregulated genes (left) or downregulated genes (right) in TSW vs MONO as defined in this work for the same cell line. TSW: MM cells in the upper transwell of a coculture with BMSCs; MONO: MM.1S cells in monoculture. BTZ: 10nm bortezomib for 8 hrs. (**B**) GSEA of expressed genes sorted by fold change of expression (CFZ/DMSO; MM.1S) from high (red) to low (blue) based on expression data from Huang et al., [7] against gene set (vertical bars) for upregulated genes (left) or downregulated genes (right) in TSW vs MONO as defined in this work for the same cell line. CFZ: 18nm carfilzomib for 24 hrs. (**C**) GSEA of expressed genes sorted by fold change of expression (BTZ-R/PRT; RPMI8226) from high (red) to low (blue) based on expression data from Bennett et al. [8] against gene set (vertical bars) for upregulated genes (left) or downregulated genes (right) in TSW vs MONO as defined in this work for the same cell line. BTZ-R: bortezomib resistance cell line; PRT: parental cell line where the resistant line is derived. (**D**) GSEA of expressed genes sorted by fold change of expression (CFZ-R/PRT; RPMI8226) from high (red) to low (blue) based on expression data from Bennett et al. [8] against gene set (vertical bars) for upregulated genes (left) or downregulated genes (right) in TSW vs MONO as defined in this work for the same cell line. CFZ-R: carfilzomib resistance cell line.

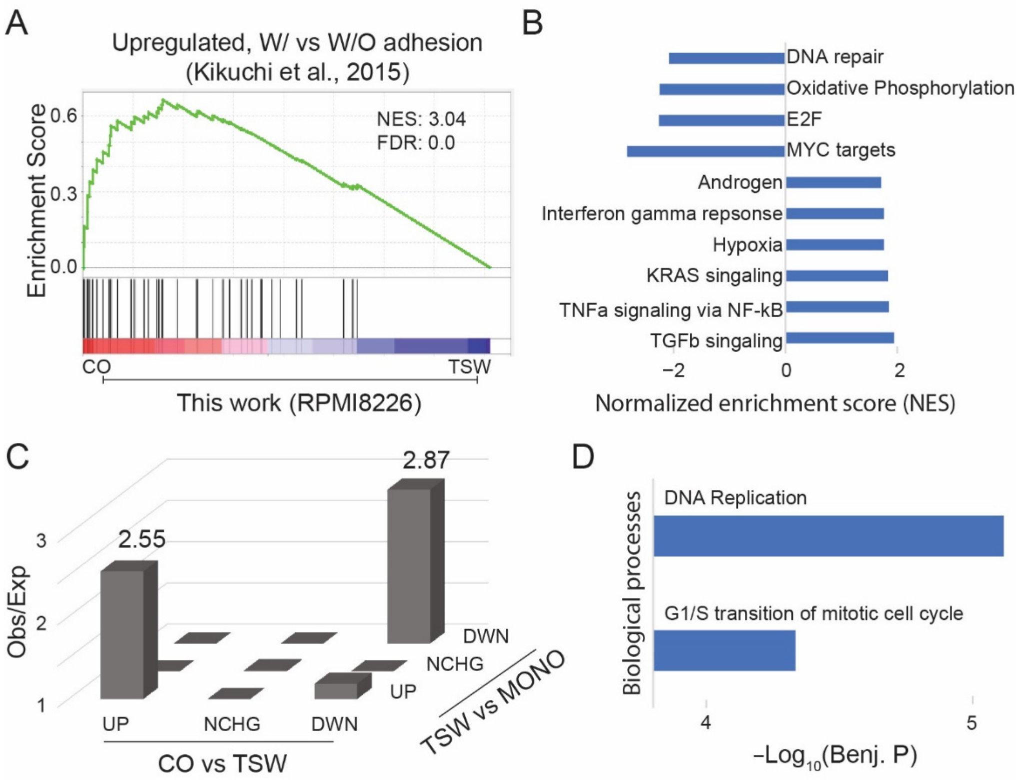

Figure S4. Characterization of transcription signatures induced by physical adhesion to BMSCs. (**A**) GSEA of expressed genes sorted by fold change of expression (CO/TSW; RPMI8226) from high (red) to low (blue) based on expression data from this work against gene set (vertical bars) for adhesion-induced genes by Kikuchi et al. [9] for the same cell line. TSW: MM cells in the upper transwell of a coculture with BMSCs; CO: MM cells in the lower chamber. NES: normalization enrichment score. (**B**) Bar plot summarizing NESs of GSEA analysis of expressed genes sorted by fold change of expression (CO/TSW; RPMI8226) from high (red) to low (blue) based on expression data from this work against MSigDB hallmark gene collection. Shown for FDR < 0.05. (**C**) 3D bar graphs for the ratios of observed (Obs) to expected (Exp) numbers of genes sorted by on their expression changes from MONO to TSW (y-axis) and from TSW to CO (x-axis): upregulated (UP), downregulated (DWN), and no change (NCHG). MONO: MM cells in monoculture. (**D**) Enriched biological processes for genes commonly down-regulated from MONO to TSW and from TSW to CO.

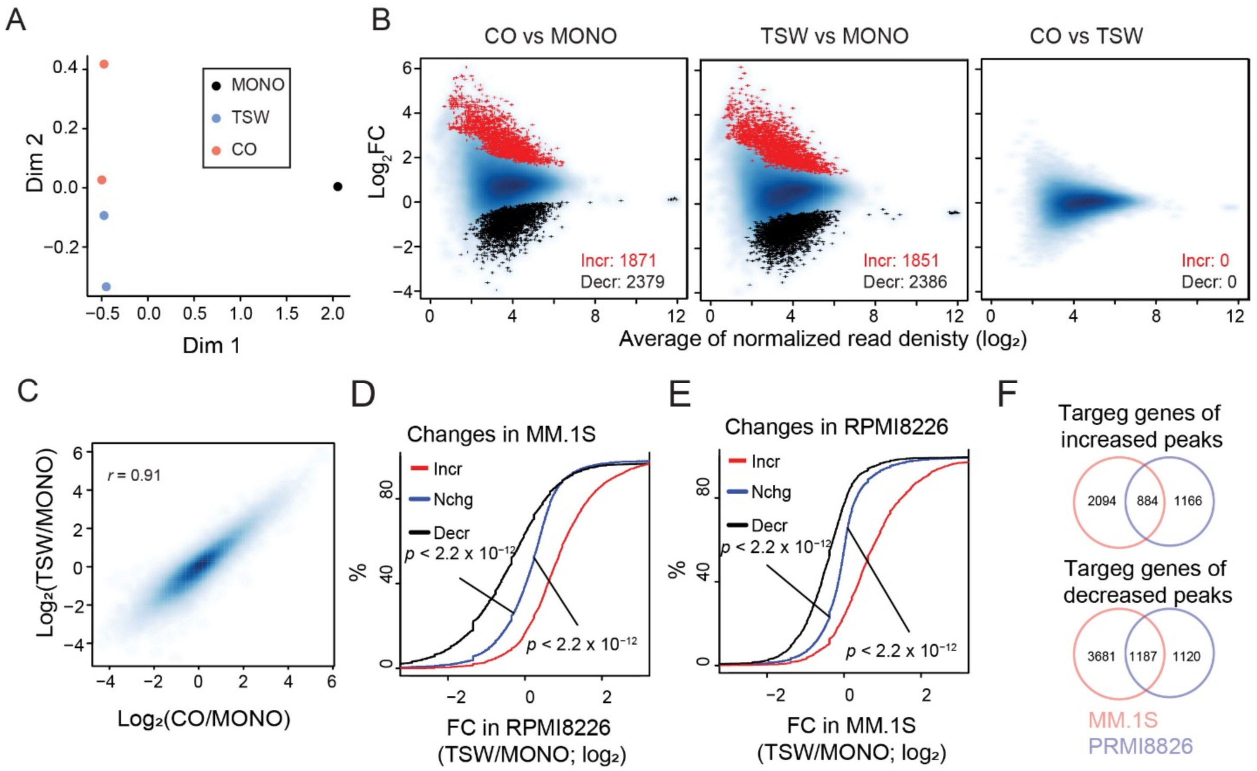

Figure S5. BMSC-induced changes in regulome observed in MM1.S are reproducible with RPMI8226. (**A**) MDS analysis of RPMI8226 samples from MONO, TSW and CO based on chromatin accessibility at reference accessible regions (peaks) defined by Omni-ATAC. (**B**) MA-plots displaying average of normalized Omni-ATAC read density (by total library size and peak length) (x-axis) and fold change of the normalized read density (y-axis) for the comparisons of CO vs MONO (left), TSW vs MONO (middle), and CO vs TSW (right) from RPMI8226 samples. Red: reference peaks increasing in accessibility (Incr); Red: reference peaks decreasing in accessibility (Decr); blue: all reference peaks for RPMI8226. (**C**) Smoothed scatter plot comparing the fold changes in accessibility at reference peaks between CO vs MONO and TSW vs MONO for RMPI8226 samples. *r*: Pearson correlation coefficient. (**D**) Empirical cumulative distribution of the fold change of chromatin accessibility (TSW/MONO) in RPMI8226 for reference peaks defined in MM.1S and sorted by increase (Incr), decrease (Decr), or no change (Nchg) in accessibility from MONO to TSW in MM.1S. A line shifting to the right indicates an overall increase in the fold change. P-value by the Kolmogorov-Smirnov (K-S) test. (**E**) Empirical cumulative distribution of the fold change of chromatin accessibility (TSW/MONO) in MM.1S for reference peaks defined in RPMI8226 and sorted by increase (Incr), decrease (Decr), or no change (Nchg) in accessibility from MONO to TSW in RPMI8226. (**F**) Venn diagram for target genes predicted by GREAT [10] for peaks that increased (upper) or decreased (lower) in accessibility as defined in MM.1S or in RPMI8226.

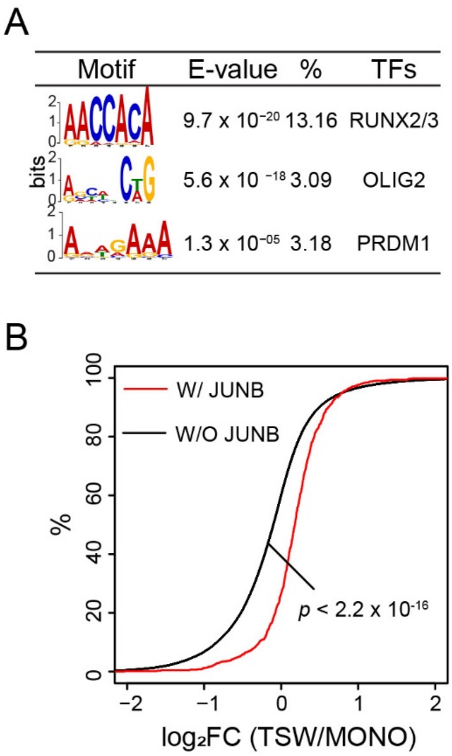

Figure S6. Distal reference peaks bound by JUNB preferentially increased chromatin accessibility in TSW vs MONO. (**A**) Top motifs discovered by MEME for peaks decreasing in accessibility from MONO to TSW, with E-value, % of peaks containing the motif, and representative TFs indicated. (**B**) Empirical cumulative distribution of the fold change of chromatin accessibility (TSW/MONO; MM.1S) of distal reference peaks sorted by their status of overlapping with JUNB ChIP-seq peaks as defined from a previous study for MM.1S stimulated with IL6 [11]. A line shifting to the right indicates an overall increase in chromatin accessibility. *P*-value by the Kolmogorov-Smirnov (K-S) test.

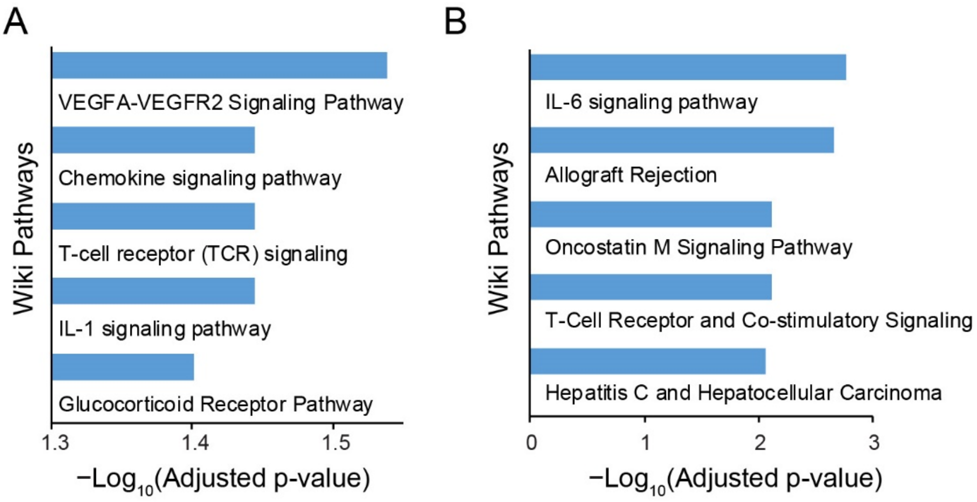

Figure S7. WikiPathways enrichment analysis for targets genes predicted for distal peaks increasing in accessibility (upper transwell vs monoculture) and containing JUNB motif (**A**) or ATF::CEBP motif (**B**).

A

Motif	%Obs	%Exp	P-value	Candidate TFs
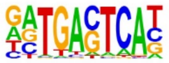	27.38%	9.13%	1 x 10^{-148}	JUNB
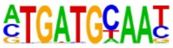	5.04.%	1.13.%	1 x 10^{-39}	ATF4/CEBPβ
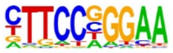	2.83%	0.87%	1 x 10^{-15}	STAT3

B

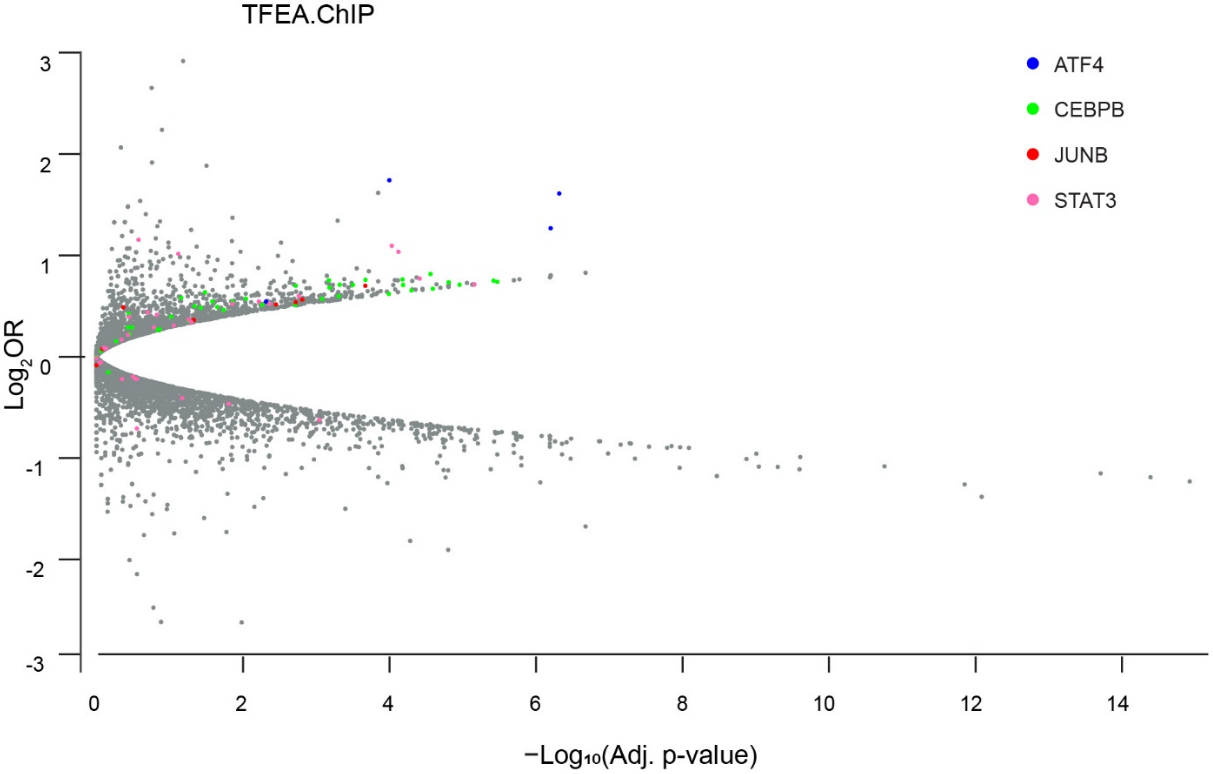

Figure S8. STAT3 motif discovered through an integrative analysis with public ChIP-seq data. (**A**) Top motifs detected by HOMER [12] for peaks increased in accessibility from MONO to TSW, with % of observed peaks containing the motif, % of expectation, and candidate TFs recognizing the motif. We used HOMER with known motifs derived from public ChIP-Seq data for the analysis. (**B**) Scatter plot for the adjusted p-value and the log-odds ratio (LOR) from through TFEA.ChIP analysis [13], which assess the significance of association of target genes defined from public ChIP datasets and genes upregulated in TSW vs MONO. Indicated are datasets corresponding to ATF4, CEBPB, JUNB, and STAT3 in difference cell systems.

Table S1. Statistics for RNA-Seq libraries.

Cell line	Culture condition *	Replica te	#Total PETs **	#Informative PETs ***
MM.1S	MONO	1	13,216,333	9,111,102
MM.1S	MONO	2	28,588,717	15,001,492
MM.1S	MONO	3	18,823,082	13,387,730
MM.1S	CO	1	21,611,316	16,320,467
MM.1S	CO	2	21,140,836	15,526,823
MM.1S	CO	3	21,096,806	14,081,583
MM.1S	TSW	1	20,402,171	14,445,033
MM.1S	TSW	2	21,343,762	15,424,277
MM.1S	TSW	3	18,511,976	13,326,916
RPMI8226	MONO	1	18,658,917	12,643,980
RPMI8226	MONO	2	22,493,124	15,001,492
RPMI8226	CO	1	23,094,305	16,583,475
RPMI8226	CO	2	28,063,118	19,149,166
RPMI8226	TSW	1	29,912,274	18,869,588
RPMI8226	TSW	2	28,335,953	18,210,518

* MONO: MM cells in monoculture; CO: MM cells in the lower chamber of coculture with BMSCs (HS5); TSW: MM cells in the upper transwell of coculture with BMSCs (HS5).
** PET: pair-end tags. Total number of PETs sequenced.
*** PETs mapped to gene transcripts annotated by RefSeq for human.

Table S2. Statistics for Omni-ATAC libraries.

Cell line	Condition *	CO	#Total PETs **	#Informative PETs ***
MM.1S	MONO	1	34,697,213	21,317,259
MM.1S	MONO	2	8,898,587	5,431,416
MM.1S	MONO	3	45,956,469	28,755,569
MM.1S	CO	1	27,611,262	17,144,945
MM.1S	CO	2	40,060,039	23,736,394
MM.1S	CO	3	31,667,256	20,367,269
MM.1S	TSW	1	38,891,754	23,809,885
MM.1S	TSW	2	41,133,656	25,398,057
MM.1S	TSW	3	36,214,417	23,135,511
RPMI8226	MONO	1	17,163,681	11,972,276
RPMI8226	CO	1	16,063,182	11,455,335
RPMI8226	CO	2	27,520,648	17,101,740
RPMI8226	TSW	1	21,978,813	15,604,470
RPMI8226	TSW	2	21,552,399	14,839,599

* MONO: MM cells in monoculture; CO: MM cells in the lower chamber of coculture with BMSCs (HS5); TSW: MM cells in the upper transwel of coculture with BMSCs (HS5).
** PET: pair-end tags. Totoal number of PETs sequenced.
*** Uniquely mapped (MAPQ score > 10) and non-redundant PETs.

Table S3. Genes with multiple (≥3) distal peaks that showed an increase in accessibility in the upper transwell vs the monoculture. The table is attached as a separated xslx file.

Table S4. Genes implied in promoting drug resistance in MM cells*.

Gene	Comments	Ref
CDK6	CDK6 overexpression in MM.1S and OPM2 reduces sensitivity to lenalidomide and pomalidomide	[14]
HIF1A	Overexpression of HIF1A in MM.1S induces resistant to bortezomib	[15]
MCL1	BMSCs or soluble factors such as IL6 and IGF-1 upregulates MCL1 expression in MM cells and confer drug resistance to bortezomib.	[16]
MITF	The siRNA silencing of MITF improves apoptotic rates of MM cells treated with dexamethasone	[17]
PPP3CA	PPP3CA knockdown in U266 cells enhances the sensitivity to bortezomib compared with control cells.	[18]
PTP4A3	IL6 induces transcriptional upregulation of PRL-3 (encoded by PTP4A3), which rephosphorylates STAT3, resulting in bortezomib resistance in multiple myeloma.	[19]
RUNX3	Inhibition of RUNX3 results in sensitization of myeloma cells to lenalidomide.	[20]
USP14	RPMI8226 cells adhered to HS-5 become more sensitive to mitoxantrone or doxorubicin apoptosis in the absence of USP14.	[21]
SGK1	Growth factors or BMSCs activate SGK1 expression in MM cells. SGK1 inhibition enhances the cytotoxic effects of bortezomib and ixazomib.	[3,22]

* Key words including "drug resistance", "myeloma", and gene name were searched in Google Scholar for each of the 49 genes listed Figure 5E. Literature review was then conducted to identify genes implied in promoting drug resistance in MM.

References

1. McMillin, D.W.; Delmore, J.; Weisberg, E.; Negri, J.M.; Geer, D.C.; Klippel, S.; Mitsiades, N.; Schlossman, R.L.; Munshi, N.C.; Kung, A.L.; et al. Tumor cell-specific bioluminescence platform to identify stroma-induced changes to anticancer drug activity. *Nat. Med.* **2010**, *16*, 483-489, doi:10.1038/nm.2112.
2. Lam, C.; Ferguson, I.D.; Mariano, M.C.; Lin, Y.T.; Murnane, M.; Liu, H.; Smith, G.A.; Wong, S.W.; Taunton, J.; Liu, J.O.; et al. Repurposing tofacitinib as an anti-myeloma therapeutic to reverse growth-promoting effects of the bone marrow microenvironment. *Haematologica* **2018**, *103*, 1218-1228, doi:10.3324/haematol.2017.174482.
3. Fagerli, U.M.; Ullrich, K.; Stuhmer, T.; Holien, T.; Kochert, K.; Holt, R.U.; Bruland, O.; Chatterjee, M.; Nogai, H.; Lenz, G.; et al. Serum/glucocorticoid-regulated kinase 1 (SGK1) is a prominent target gene of the transcriptional response to cytokines in multiple myeloma and supports the growth of myeloma cells. *Oncogene* **2011**, *30*, 3198-3206, doi:10.1038/onc.2011.79.
4. Brocke-Heidrich, K.; Kretzschmar, A.K.; Pfeifer, G.; Henze, C.; Loffler, D.; Koczan, D.; Thiesen, H.J.; Burger, R.; Gramatzki, M.; Horn, F. Interleukin-6-dependent gene expression profiles in multiple myeloma INA-6 cells reveal a Bcl-2 family-independent survival pathway closely associated with Stat3 activation. *Blood* **2004**, *103*, 242-251, doi:10.1182/blood-2003-04-1048.
5. Croonquist, P.A.; Linden, M.A.; Zhao, F.; Van Ness, B.G. Gene profiling of a myeloma cell line reveals similarities and unique signatures among IL-6 response, N-ras-activating mutations, and coculture with bone marrow stromal cells. *Blood* **2003**, *102*, 2581-2592, doi:10.1182/blood-2003-04-1227.
6. Le Moigne, R.; Aftab, B.T.; Djakovic, S.; Dhimolea, E.; Valle, E.; Murnane, M.; King, E.M.; Soriano, F.; Menon, M.K.; Wu, Z.Y.; et al. The p97 Inhibitor CB-5083 Is a Unique Disrupter of Protein Homeostasis in Models of Multiple Myeloma. *Mol. Cancer Ther.* **2017**, *16*, 2375-2386, doi:10.1158/1535-7163.MCT-17-0233.
7. Huang, H.H.; Ferguson, I.D.; Thornton, A.M.; Bastola, P.; Lam, C.; Lin, Y.H.T.; Choudhry, P.; Mariano, M.C.; Marcoulis, M.D.; Teo, C.F.; et al. Proteasome inhibitor-induced modulation reveals the spliceosome as a specific therapeutic vulnerability in multiple myeloma. *Nature Communications* **2020**, *11*, doi:ARTN 1931
8. Bennett, M.K.; Li, M.; Tea, M.N.; Pitman, M.R.; Toubia, J.; Wang, P.P.; Anderson, D.; Creek, D.J.; Orlowski, R.Z.; Gliddon, B.L.; et al. Resensitising proteasome inhibitor-resistant myeloma with sphingosine kinase 2 inhibition. *Neoplasia* **2022**, *24*, 1-11, doi:10.1016/j.neo.2021.11.009.
9. Kikuchi, J.; Koyama, D.; Wada, T.; Izumi, T.; Hofgaard, P.O.; Bogen, B.; Furukawa, Y. Phosphorylation-mediated EZH2 inactivation promotes drug resistance in multiple myeloma. *J. Clin. Invest.* **2015**, *125*, 4375-4390, doi:10.1172/JCI80325.
10. McLean, C.Y.; Bristor, D.; Hiller, M.; Clarke, S.L.; Schaar, B.T.; Lowe, C.B.; Wenger, A.M.; Bejerano, G. GREAT improves functional interpretation of cis-regulatory regions. *Nat. Biotechnol.* **2010**, *28*, 495-501, doi:10.1038/nbt.1630.

11. Fan, F.J.; Malvestiti, S.; Vallet, S.; Lind, J.; Garcia-Manteiga, J.M.; Morelli, E.; Jiang, Q.Y.; Seckinger, A.; Hose, D.; Goldschmidt, H.; et al. JunB is a key regulator of multiple myeloma bone marrow angiogenesis. *Leukemia* **2021**, *35*, 3509-3525, doi:10.1038/s41375-021-01271-9.
12. Heinz, S.; Benner, C.; Spann, N.; Bertolino, E.; Lin, Y.C.; Laslo, P.; Cheng, J.X.; Murre, C.; Singh, H.; Glass, C.K. Simple combinations of lineage-determining transcription factors prime cis-regulatory elements required for macrophage and B cell identities. *Mol. Cell* **2010**, *38*, 576-589, doi:10.1016/j.molcel.2010.05.004.
13. Puente-Santamaria, L.; Wasserman, W.W.; Del Peso, L. TFEA.ChIP: a tool kit for transcription factor binding site enrichment analysis capitalizing on ChIP-seq datasets. *Bioinformatics* **2019**, *35*, 5339-5340, doi:10.1093/bioinformatics/btz573.
14. Ng, Y.L.D.; Bohl, S.; Ramberger, E.; Popp, O.; Bauhuf, I.; Dolnik, A.; Steinebach, C.; Gutschow, M.; Bullinger, L.; Mertins, P.; et al. Quantitative Proteomic Analysis of Relapsed Multiple Myeloma Identifies CDK6 Upregulation As a Potential Targetable Resistance Mechanism for Lenalidomide. *Blood* **2019**, *134*, doi:10.1182/blood-2019-130682.
15. Maiso, P.; Huynh, D.; Moschetta, M.; Sacco, A.; Aljawai, Y.; Mishima, Y.; Asara, J.M.; Roccaro, A.M.; Kimmelman, A.C.; Ghobrial, I.M. Metabolic Signature Identifies Novel Targets for Drug Resistance in Multiple Myeloma. *Cancer Res.* **2015**, *75*, 2071-2082, doi:10.1158/0008-5472.Can-14-3400.
16. Pei, X.Y.; Dai, Y.; Felthousen, J.; Chen, S.; Takabatake, Y.; Zhou, L.; Youssefian, L.E.; Sanderson, M.W.; Bodie, W.W.; Kramer, L.B.; et al. Circumvention of Mcl-1-dependent drug resistance by simultaneous Chk1 and MEK1/2 inhibition in human multiple myeloma cells. *PLoS ONE* **2014**, *9*, e89064, doi:10.1371/journal.pone.0089064.
17. Zhang, B.P.; Ma, L.; Wei, J.; Hu, J.Y.; Zhao, Z.C.; Wang, Y.P.; Chen, Y.; Zhao, F. miR-137 Suppresses the Phosphorylation of AKT and Improves the Dexamethasone Sensitivity in Multiple Myeloma Cells Via Targeting MITF. *Curr. Cancer Drug Tar.* **2016**, *16*, 807-817, doi:10.2174/1568009616666160203114140.
18. Imai, Y.; Ohta, E.; Takeda, S.; Sunamura, S.; Ishibashi, M.; Tamura, H.; Wang, Y.H.; Deguchi, A.; Tanaka, J.; Maru, Y.; et al. Histone deacetylase inhibitor panobinostat induces calcineurin degradation in multiple myeloma. *JCI Insight* **2016**, *1*, e85061, doi:10.1172/jci.insight.85061.
19. Chong, P.S.Y.; Zhou, J.B.; Lim, J.S.L.; Hee, Y.T.; Chooi, J.Y.; Chung, T.H.; Tan, Z.T.; Zeng, Q.; Waller, D.D.; Sebag, M.; et al. IL6 Promotes a STAT3-PRL3 Feedforward Loop via SHP2 Repression in Multiple Myeloma. *Cancer Res.* **2019**, *79*, 4679-4688, doi:10.1158/0008-5472.Can-19-0343.
20. Zhou, N.; Gutierrez-Uzquiza, A.; Zheng, X.Y.; Chang, R.X.; Vogl, D.T.; Garfall, A.L.; Bernabei, L.; Saraf, A.; Florens, L.; Washburn, M.P.; et al. RUNX proteins desensitize multiple myeloma to lenalidomide via protecting IKZFs from degradation. *Leukemia* **2019**, *33*, 2006-2021, doi:10.1038/s41375-019-0403-2.
21. Xu, X.H.; Liu, J.; Shen, C.Y.; Ding, L.L.; Zhong, F.; Ouyang, Y.; Wang, Y.C.; He, S. The role of ubiquitin-specific protease 14 (USP14) in cell adhesion-mediated drug resistance (CAM-DR) of multiple myeloma cells. *Eur. J. Haematol* **2017**, *98*, 4-12, doi:10.1111/ejh.12729.
22. Tsubaki, M.; Takeda, T.; Matsuda, T.; Seki, S.; Tomonari, Y.; Koizumi, S.; Nagatakiya, M.; Katsuyama, M.; Yamamoto, Y.; Tsurushima, K.; et al. Activation of Serum/Glucocorticoid Regulated Kinase 1/Nuclear Factor-kappaB Pathway Are Correlated with Low Sensitivity to Bortezomib and Ixazomib in Resistant Multiple Myeloma Cells. *Biomedicines* **2021**, *9*, doi:10.3390/biomedicines9010033.
